# Stereotactic Body Radiotherapy as a Salvage Treatment for Regional Lymph Node Recurrence of Cervical Cancer: Two Case Reports With Complete Metabolic Response and Literature Review

**DOI:** 10.7759/cureus.111361

**Published:** 2026-06-23

**Authors:** Amina Majdi, Sara Harbaj, Boutaina Agdi, Rania Chakir, Lachgar Amine, Karima Nouni, Hanane Elkacemi, Tayeb Kebdani, Khalid Hassouni

**Affiliations:** 1 Department of Radiation Oncology, National Institute of Oncology, Rabat, MAR; 2 Faculty of Medicine, Mohammed V University, Rabat, MAR; 3 Department of Radiotherapy, National Institute of Oncology, Rabat, MAR; 4 Faculty of Medicine and Pharmacy, Mohammed V University, Rabat, MAR

**Keywords:** cervical cancer, local control, lymph node metastases, nodal recurrence, oligometastatic disease, stereotactic body radiotherapy

## Abstract

Locoregional and regional nodal recurrence remains a major therapeutic challenge in cervical cancer, particularly in patients previously treated with pelvic radiotherapy, where salvage options are limited by cumulative toxicity to surrounding organs at risk. In selected patients with limited-volume recurrent disease, stereotactic body radiotherapy (SBRT) has emerged as a highly conformal salvage approach capable of delivering ablative radiation doses while minimizing exposure to adjacent normal tissues.

We report two cases of limited-volume regional nodal recurrence from cervical squamous cell carcinoma successfully managed with SBRT. The first patient, a 32-year-old woman initially diagnosed with International Federation of Gynecology and Obstetrics (FIGO) 2018 stage IIIC1 disease, developed multifocal pelvic and para-aortic lymph node recurrence 12 months after completion of definitive treatment. The second patient, a 48-year-old woman with FIGO 2018 stage IIB disease, presented with isolated para-aortic nodal recurrence after a 33-month disease-free interval. In both cases, recurrence was identified by fluorine-18 fluorodeoxyglucose positron emission tomography-computed tomography (^18^F-FDG PET-CT) and discussed in a multidisciplinary tumor board. Histological confirmation was not obtained because of the deep location of the lesions, their proximity to major vascular structures, the procedural risks of biopsy, and the highly suggestive PET-CT findings in patients with previous cervical squamous cell carcinoma. Salvage surgery was considered technically challenging because of previous irradiation and fibrosis. SBRT was delivered using volumetric modulated arc therapy (VMAT) with daily image guidance. The first patient received 40 Gy in five fractions for pelvic and para-aortic nodal recurrence, whereas the second received 30 Gy in three fractions for isolated para-aortic nodal recurrence. Treatment was well tolerated, with no acute grade ≥2 toxicity and no late grade ≥3 toxicity observed. Follow-up PET-CT demonstrated a complete metabolic response in both patients. At the last follow-up, the first patient remained disease-free 38 months after SBRT, and the second patient remained disease-free 40 months after SBRT, without evidence of progression or significant late toxicity.

SBRT appears to be a feasible and well-tolerated salvage option for selected patients with limited-volume regional nodal recurrence of cervical cancer after prior definitive chemoradiotherapy. PET-CT-guided SBRT allows delivery of ablative doses with durable local control while limiting toxicity to previously irradiated normal tissues. These findings support further prospective evaluation of curative-intent local treatment strategies in this setting.

## Introduction

Cervical cancer remains one of the leading causes of cancer-related morbidity and mortality among women worldwide, particularly in low- and middle-income countries where access to screening programs and human papillomavirus (HPV) vaccination remains heterogeneous [1]. Despite significant advances in concurrent chemoradiotherapy (CCRT), image-guided brachytherapy, and modern imaging-based staging, recurrence continues to represent a major therapeutic challenge, especially in patients with locally advanced disease [1,2]. Regional lymph node recurrence involving pelvic, common iliac, or para-aortic nodal stations is a frequent pattern of failure and is associated with poor outcomes when effective salvage treatment cannot be achieved [2].

Management of regional nodal recurrence after prior pelvic irradiation remains particularly challenging. Salvage surgery may provide durable local control in selected patients but is often technically difficult because of radiation-induced fibrosis, altered anatomy, and proximity to major vascular structures [3]. Systemic therapy remains the standard treatment for disseminated recurrent disease; however, in patients with isolated or limited regional relapse, the optimal role of local ablative approaches remains an area of active investigation [4]. Conventional re-irradiation using standard fractionation is similarly constrained by cumulative doses to surrounding organs at risk (OARs), including the bowel, kidneys, bladder, spinal canal, and cauda equina [5].

The concepts of oligometastatic and oligorecurrent disease, initially proposed by Hellman and Weichselbaum, suggest that selected patients with limited-volume recurrence may benefit from aggressive local treatment with curative intent rather than exclusively palliative systemic therapy [6]. Subsequent consensus recommendations from the European Society for Radiotherapy and Oncology (ESTRO) and the European Organisation for Research and Treatment of Cancer (EORTC) further refined the classification of oligometastatic disease and emphasized the role of metastasis-directed therapies in appropriately selected patients [5]. In cervical cancer, increasing evidence indicates that patients with isolated regional nodal recurrence may achieve durable disease control following local ablative treatment strategies, including stereotactic body radiotherapy (SBRT) [7-9].

SBRT combines advanced image guidance, highly conformal treatment planning, and steep dose gradients, enabling delivery of ablative radiation doses while minimizing exposure to adjacent normal tissues [10,11]. Beyond direct tumor cell killing through DNA damage, high-dose hypofractionated irradiation induces endothelial apoptosis, microvascular dysfunction, and tumor necrosis, mechanisms that may contribute to overcoming radioresistance in hypoxic tumor regions [10,11]. In addition, preclinical and translational studies have demonstrated that SBRT may enhance anti-tumor immune responses through immunogenic cell death, tumor antigen release, and activation of cytotoxic T-lymphocytes [12].

Several retrospective series have reported encouraging local control rates ranging from 70% to 90% following SBRT for nodal recurrences of gynecological malignancies, including patients previously treated with pelvic irradiation [2,7-9,13]. Furthermore, SBRT has demonstrated acceptable toxicity profiles when modern image-guided radiotherapy techniques and strict OAR constraints are applied [14].

Current international guidelines and consensus recommendations support individualized curative-intent treatment strategies for selected patients presenting with isolated regional nodal recurrence or limited-volume recurrent disease without evidence of disseminated progression [1,5,15]. In this setting, local ablative therapies such as SBRT may represent an alternative to surgery or systemic therapy alone following multidisciplinary evaluation. The role of SBRT is particularly relevant for para-aortic or pelvic nodal recurrences, where durable local control may be achievable while avoiding the morbidity associated with extensive salvage surgery or repeated systemic treatment [1,5,15].

Although prospective cervical cancer-specific data remain limited, the randomized phase II SABR-COMET trial demonstrated improved overall survival and progression-free survival with the addition of stereotactic ablative radiotherapy in selected patients with oligometastatic disease across multiple tumor types, supporting the broader concept of metastasis-directed therapy [16,17].

Herein, we report two cases of limited-volume regional nodal recurrence of cervical squamous cell carcinoma (SCC) successfully treated with positron emission tomography-computed tomography (PET-CT)-guided SBRT after prior definitive chemoradiotherapy. These cases highlight the feasibility of salvage SBRT in previously irradiated patients, its favorable dosimetric characteristics, and its potential to achieve durable metabolic response and long-term disease control with limited toxicity.

## Case presentation

Case 1

A 32-year-old woman with no significant past medical history was diagnosed in March 2021 with biopsy-proven SCC of the cervix. Baseline pelvic MRI performed in March 2021 demonstrated a cervical mass measuring 46 × 27 × 42 mm associated with a 12 mm left external iliac lymph node, consistent with regional nodal involvement (International Federation of Gynecology and Obstetrics (FIGO) 2018 stage IIIC1).

The patient underwent definitive CCRT consisting of pelvic external beam radiotherapy delivered to 46 Gy in 23 fractions, followed by a sequential boost of 20 Gy to the involved lymph node. Concurrent weekly cisplatin (40 mg/m²) was administered for four cycles. Treatment was completed with high-dose-rate intracavitary brachytherapy delivering 7 Gy in four fractions. The entire treatment course was completed in July 2021, without major treatment-related complications.

Post-treatment pelvic MRI performed in November 2021 demonstrated complete radiological regression of the primary cervical lesion. A residual necrotic left external iliac lymph node measuring 13 mm remained visible, with no evidence of additional disease. The patient subsequently entered a program of routine clinical and radiological surveillance.

**Figure 1:**
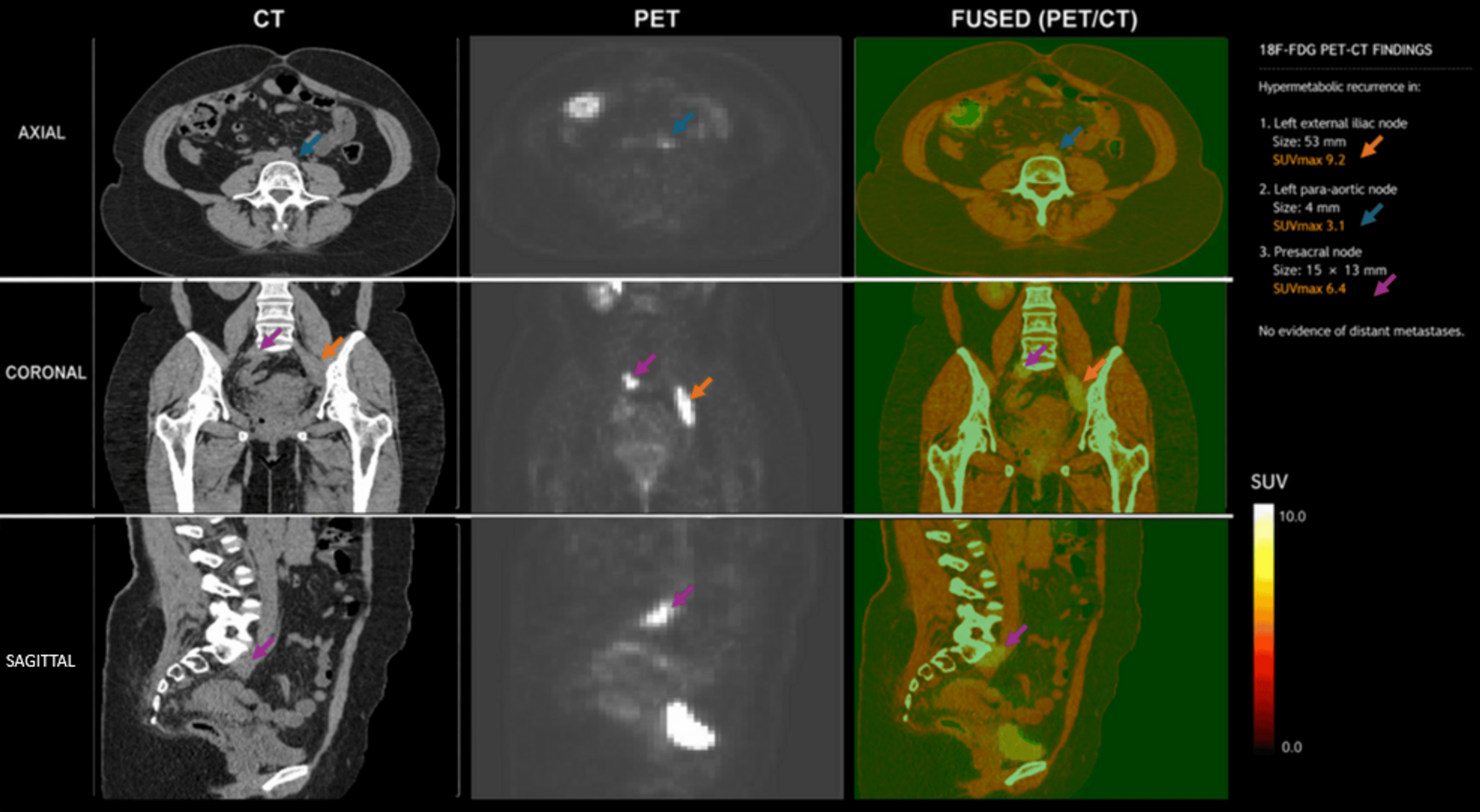
Diagnostic PET-CT of Patient 1 showing pelvic and para-aortic nodal recurrence. Hypermetabolic left external iliac (orange arrow), para-aortic (blue arrow), and presacral (purple arrow) lymph nodes were identified without evidence of distant disease. PET-CT: positron emission tomography-computed tomography; SUVmax: maximum standardized uptake value; ^18^F-FDG: fluorine-18 fluorodeoxyglucose

Twelve months after completion of treatment, the patient developed progressive lumbar pain without neurological deficit. Restaging fluorine-18 fluorodeoxyglucose (^18^F-FDG) PET-CT revealed regional nodal recurrence involving a left external iliac lymph node measuring 53 mm (maximum standardized uptake value (SUVmax) = 9.2), a left para-aortic lymph node measuring 4 mm (SUVmax = 3.1), and a presacral lymph node measuring 15 × 13 mm (SUVmax = 6.4), without evidence of visceral metastases or disseminated disease, as illustrated in the diagnostic multiplanar PET-CT images presented in Figure 1.

Histological confirmation was discussed during multidisciplinary review but was not pursued because of the deep anatomical location of the lesions, their proximity to major vascular structures, the procedural risks associated with biopsy, and the highly suggestive PET-CT findings, supporting a high clinical probability of recurrence in a patient with previously treated cervical SCC.

**Figure 2:**
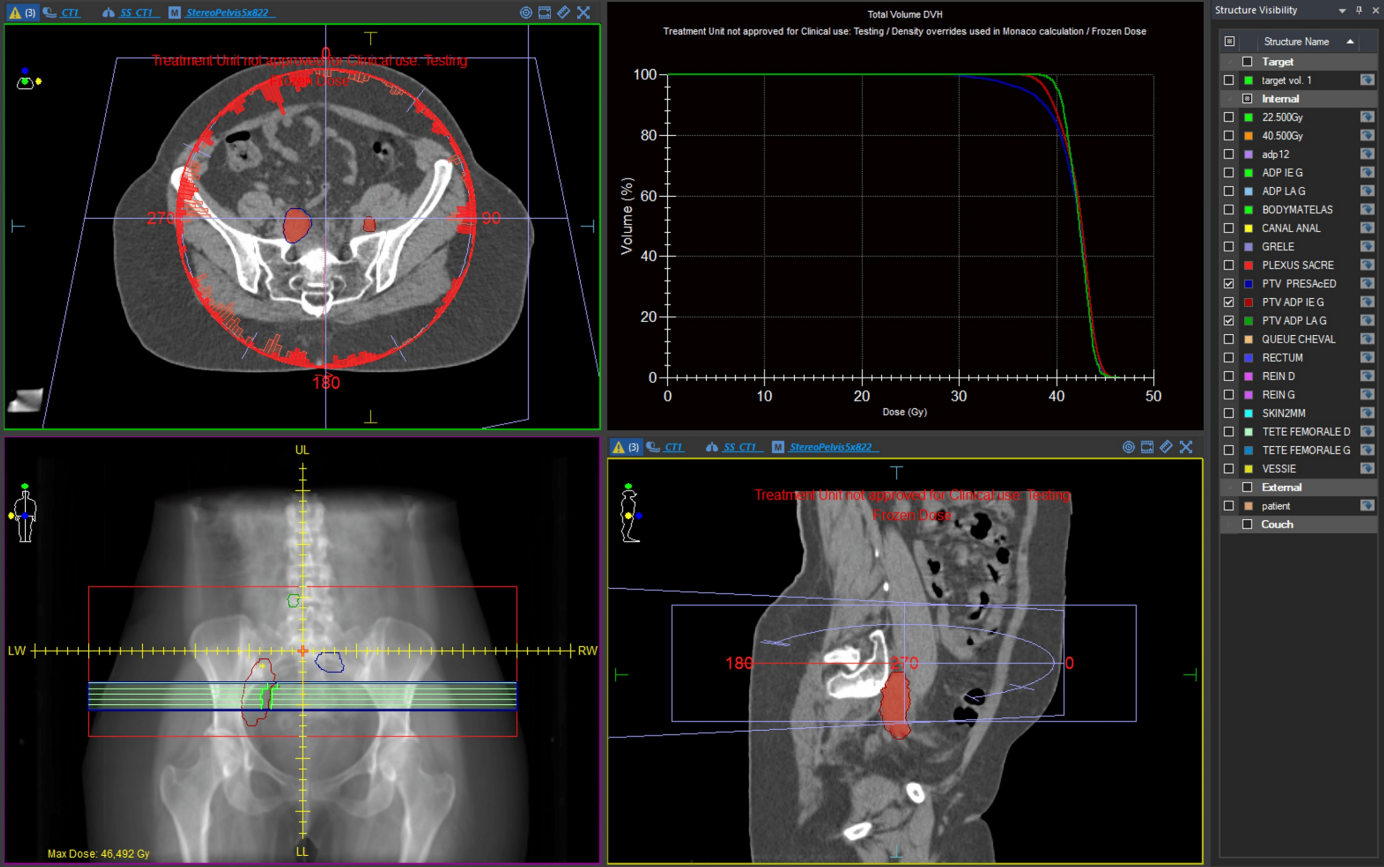
SBRT treatment plan for Patient 1 illustrating dose distribution and PTV coverage. Axial, coronal, and sagittal views demonstrating VMAT-based SBRT for pelvic and para-aortic nodal recurrence with adequate target coverage and organ-at-risk sparing. SBRT: stereotactic body radiotherapy; PTV: planning target volume; VMAT: volumetric modulated arc therapy

Following multidisciplinary tumor board discussion, salvage SBRT was selected as the preferred treatment strategy because surgical resection was considered technically challenging in the setting of prior pelvic irradiation and multifocal regional nodal recurrence. No chemotherapy, immunotherapy, targeted therapy, or other systemic treatment was administered before or after SBRT. The patient was therefore treated with SBRT alone, delivered to a total dose of 40 Gy in five fractions, followed by active surveillance. The corresponding treatment plan is shown in Figure 2.

Treatment was completed without interruption and was well tolerated, with no acute toxicity ≥ grade 2 according to the Common Terminology Criteria for Adverse Events (CTCAE) criteria. Six months after SBRT, follow-up ^18^F-FDG PET-CT demonstrated a complete metabolic response, characterized by complete disappearance of pathological FDG uptake within the treated lesions and normalization of metabolic activity to background levels (SUVmax < 2.0). At the most recent follow-up in October 2025, corresponding to 38 months after SBRT, the patient remained disease-free with sustained local control and no evidence of regional or distant recurrence.

Case 2

A 48-year-old woman with no significant medical history was diagnosed in June 2019 with biopsy-proven SCC of the cervix after presenting with abnormal vaginal bleeding and pelvic pain. Baseline pelvic MRI demonstrated a cervical tumor with parametrial extension, without pelvic sidewall involvement, consistent with FIGO 2018 stage IIB disease. Initial staging investigations revealed no regional nodal involvement or distant metastatic disease.

The patient underwent definitive CCRT consisting of pelvic external beam radiotherapy delivered to 46 Gy in 23 fractions with concurrent weekly cisplatin (40 mg/m²) for four cycles, followed by high-dose-rate intracavitary brachytherapy delivering 7 Gy × 4 fractions. Treatment was completed in September 2019 without major treatment-related toxicity.

Post-treatment clinical examination and pelvic MRI demonstrated complete regression of the primary cervical tumor with no evidence of residual pelvic disease. The patient was maintained on routine surveillance including periodic gynecological examinations and imaging studies.

**Figure 3:**
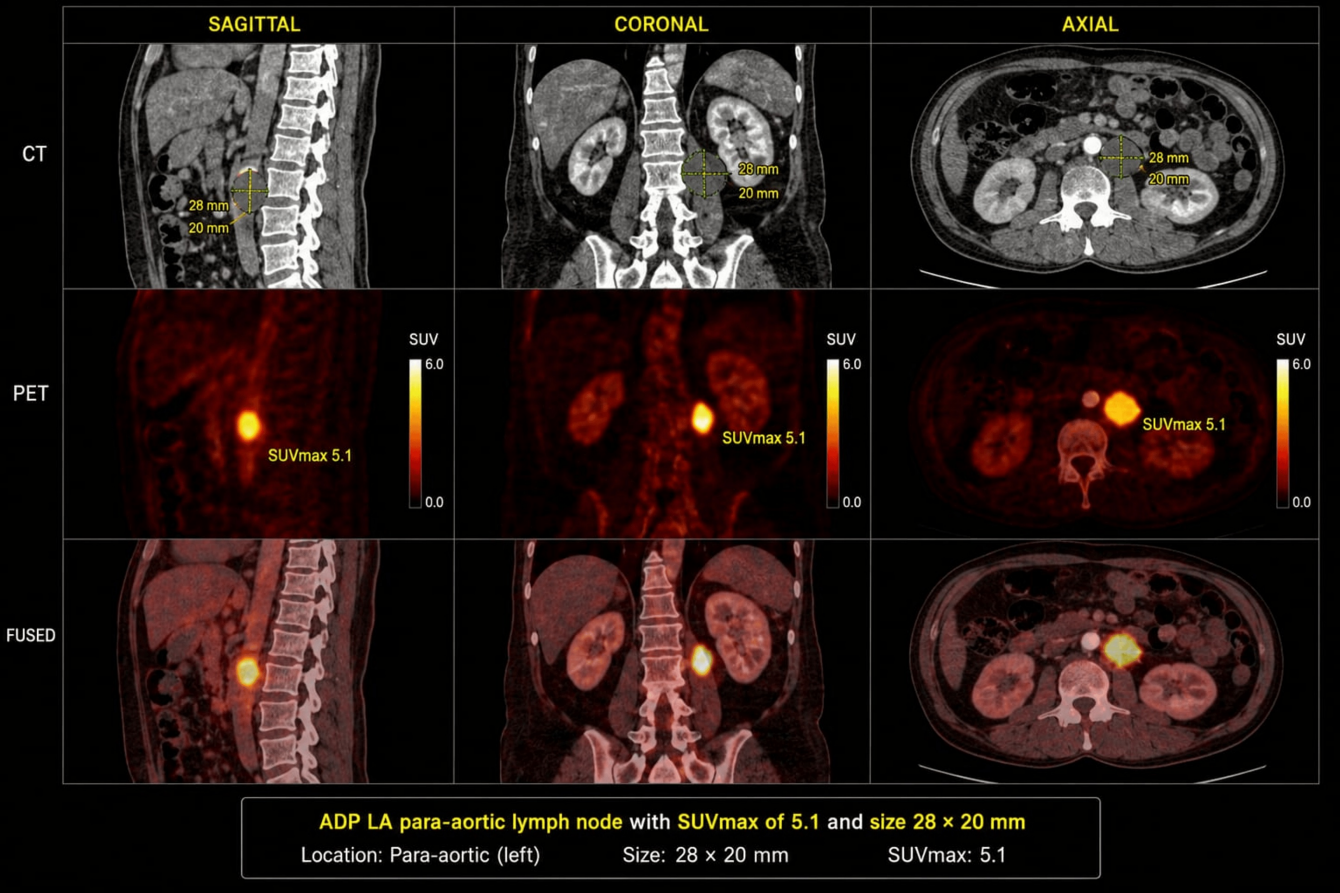
Diagnostic PET-CT of Patient 2 showing isolated para-aortic nodal recurrence. A solitary hypermetabolic para-aortic lymph node was identified without evidence of additional regional or distant disease. PET-CT: positron emission tomography-computed tomography; SUVmax: maximum standardized uptake value

After approximately 33 months of disease-free follow-up, surveillance ^18^F-FDG PET-CT performed in June 2022 revealed a solitary hypermetabolic left para-aortic lymph node measuring 28 × 20 mm with a SUVmax = 5.1, highly suggestive of isolated regional nodal recurrence, without evidence of pelvic recurrence, visceral metastases, or other distant disease, as demonstrated in the diagnostic images in Figure 3.

Histological confirmation was discussed during multidisciplinary review but was not pursued because of the deep anatomical location of the lesion, its close proximity to major vascular structures, and the PET-CT findings supporting a high clinical probability of recurrence in a patient with a previous history of cervical SCC.

The case was reviewed at a multidisciplinary tumor board involving radiation oncologists, gynecologic oncologists, radiologists, nuclear medicine physicians, and medical oncologists. Salvage para-aortic lymphadenectomy was initially considered because of the isolated nature of the recurrence. However, intraoperative exploration revealed extensive post-radiation retroperitoneal fibrosis with intimate adherence of the lymph node to the abdominal aorta and adjacent vascular structures, resulting in abandonment of the surgical procedure because of the high risk of major vascular injury.

**Figure 4:**
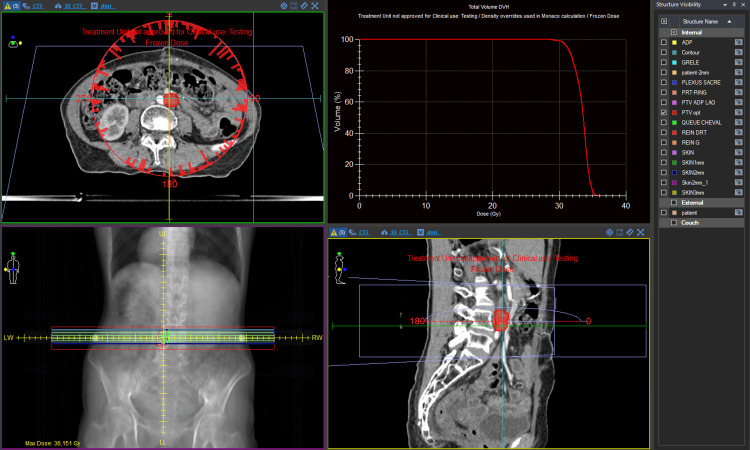
SBRT treatment plan for Patient 2 illustrating dose distribution and PTV coverage. Axial, coronal, and sagittal views demonstrating VMAT-based SBRT for isolated para-aortic nodal recurrence with conformal target coverage and preservation of adjacent organs at risk. SBRT: stereotactic body radiotherapy; PTV: planning target volume; VMAT: volumetric modulated arc therapy

Given the absence of disseminated disease and the technical limitations of surgical salvage, SBRT was selected as the preferred treatment approach following multidisciplinary review. No chemotherapy, immunotherapy, targeted therapy, or other systemic treatment was administered before or after SBRT. The patient was therefore treated with SBRT alone, delivered in July 2022 to a total dose of 30 Gy in three fractions using VMAT, followed by routine clinical and radiological surveillance. The corresponding treatment plan is shown in Figure 4.

Treatment was completed without interruption and was well tolerated, with no acute toxicity ≥ grade 2 according to CTCAE criteria. Follow-up ^18^F-FDG PET-CT performed three months after SBRT demonstrated complete metabolic response, with complete resolution of FDG uptake within the treated para-aortic lymph node and normalization of metabolic activity to background levels (SUVmax < 2.0).

At the most recent follow-up evaluation in November 2025, corresponding to 40 months after SBRT, the patient remained clinically and radiologically disease-free, with sustained local control and no evidence of regional or distant progression. Mild late gastrointestinal toxicity consisting of intermittent grade 1 diarrhea was reported and managed conservatively with dietary measures and symptomatic treatment.

Radiotherapy planning and dosimetry

Both patients underwent CT simulation in the supine position using a customized vacuum immobilization mattress (Vac-Lok® type system; Civco Medical Solutions, Orange City, IA, USA) to ensure reproducible positioning and minimize setup uncertainties. A bowel preparation protocol was implemented, consisting of bowel emptying before simulation and treatment sessions. Mild laxatives or micro-enemas were prescribed when clinically indicated to reduce bowel distension and improve treatment reproducibility. Diagnostic ^18^F-FDG PET-CT images were rigidly co-registered with the planning CT scans to enhance target delineation accuracy.

The gross tumor volume (GTV) was defined as the FDG-avid lymph nodes identified on PET-CT imaging. A uniform 5 mm margin was applied to generate the planning target volume (PTV), accounting for setup variability and internal organ motion.

Treatment planning was performed using the Monaco® Treatment Planning System version 6.1.4.0 (Elekta AB, Stockholm, Sweden). SBRT was delivered on a Versa HD™ linear accelerator (Elekta AB) equipped with the latest-generation Agility™ multileaf collimator (Elekta AB). Volumetric modulated arc therapy (VMAT) was used to achieve highly conformal dose distributions with steep dose gradients around the target volumes (Figures 2, 4).

Patient positioning and setup verification were performed using the integrated iViewGT electronic portal imaging device and the XVI (X-ray Volume Imaging) platform (Elekta AB). Image guidance included two-dimensional kilovoltage (2D kV) imaging, three-dimensional cone-beam CT (3D CBCT), and four-dimensional CBCT (Symmetry™), allowing accurate target localization before treatment and intrafraction monitoring during irradiation.

Patient-specific quality assurance (QA) was performed for each SBRT plan prior to treatment delivery using the PTW Octavius® 4D system (PTW-Freiburg, Germany), comprising the Octavius® Rotation Unit Modular and the Octavius® Detector 1600 SRS. This detector array, specifically designed for small-field dosimetry, provides a detector spacing of 2.5 mm in the central region and a maximum measurement field size of 15 × 15 cm². Measured and calculated dose distributions were compared using VeriSoft® software (PTW-Freiburg). Gamma index analysis was performed using 3%/2 mm criteria with a minimum passing rate of 95%, confirming the accuracy of dose calculation and treatment delivery.

Dosimetric plan evaluation included assessment of target coverage (V100%), near-maximum dose (D2%), homogeneity index (HI), and gradient index (GI). Across both treatment plans, HI values ranged from 1.10 to 1.21 and GI values from 1.13 to 1.50, indicating acceptable dose homogeneity and sharp dose fall-off characteristics consistent with high-quality SBRT delivery.

**Figure 5:**
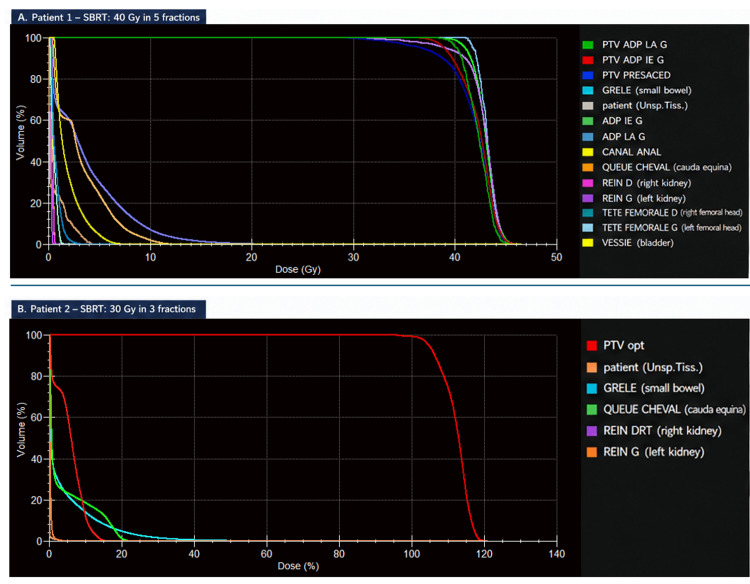
Dose-volume histograms (DVHs) confirming adherence to dose constraints and target coverage during stereotactic body radiotherapy (SBRT). (A) Patient 1: pelvic and para-aortic regional nodal recurrence treated with 40 Gy in 5 fractions. PTV V100 ranged from 93.36% to 100%, demonstrating adequate target coverage with acceptable sparing of organs at risk. (B) Patient 2: isolated para-aortic regional nodal recurrence treated with 30 Gy in 3 fractions. PTV V100 was 99.19%, demonstrating excellent target coverage with acceptable sparing of adjacent organs at risk. PTV: planning target volume

Dosimetric analysis further confirmed adequate target coverage while respecting all predefined OAR constraints, including those for the small bowel, kidneys, bladder, spinal canal, cauda equina, and femoral heads when applicable. Dose-volume histogram (DVH) evaluation demonstrated highly conformal dose distributions with steep dose gradients and acceptable cumulative OAR exposure despite prior pelvic irradiation, as illustrated in Figure 5. Detailed dosimetric parameters, plan quality indices, and OAR dose metrics are summarized in Tables 1, 2.

**Table 1:** Dosimetric parameters and OAR metrics for Patient 1. Dosimetric summary of SBRT delivered for multifocal pelvic and para-aortic regional nodal recurrence. Target coverage was considered acceptable when ≥90% of the PTV received the prescribed dose. Plan quality assessment, including D2%, homogeneity index (HI), and gradient index (GI), confirmed satisfactory target coverage, favorable dose homogeneity, and rapid dose fall-off, with all organ-at-risk (OAR) dose constraints remaining within accepted SBRT tolerance limits. SBRT: stereotactic body radiotherapy; PTV: planning target volume

Structure	Parameter evaluated	Achieved dosimetric value	SBRT constraint status
PTV green (left para-aortic lymph node)	V100% (target volume)	100%	Optimal target coverage
	D2%	44.50 Gy	Acceptable
	HI	1.10	Acceptable homogeneity
	GI	1.13	Favorable dose gradient
PTV red (left external iliac lymph node)	V100% (target volume)	98.83%	Optimal target coverage
	D2%	44.93 Gy	Acceptable
	HI	1.14	Acceptable homogeneity
	GI	1.25	Favorable dose gradient
PTV blue (presacral lymph node)	V100% (target volume)	93.36%	Acceptable target coverage
	D2%	44.78 Gy	Acceptable
	HI	1.21	Acceptable homogeneity
	GI	1.50	Favorable dose gradient
Small bowel	Dmax (absolute max)	28.2 Gy	Within SBRT tolerance (<35 Gy)
Bladder	Dmax (absolute max)	8.25 Gy	Within SBRT tolerance (<38 Gy)
Cauda equina	Dmax (absolute max)	12.48 Gy	Within SBRT tolerance (<32 Gy)
Left kidney	Mean dose	0.27 Gy	Within SBRT tolerance (<10 Gy)
Right kidney	Mean dose	0.44 Gy	Within SBRT tolerance (<10 Gy)
Left femoral head	Dmax (absolute max)	3.68 Gy	Within SBRT tolerance (<30 Gy)
Right femoral head	Dmax (absolute max)	1.92 Gy	Within SBRT tolerance (<30 Gy)

**Table 2:** Dosimetric parameters and OAR dose metrics for Patient 2. PTV coverage and organ-at-risk (OAR) dose metrics for SBRT delivered to an isolated para-aortic regional nodal recurrence. Optimal target coverage was defined as ≥95% of the PTV receiving the prescribed dose. Additional dosimetric quality indices (D2%, HI, and GI) confirmed excellent target coverage, acceptable dose homogeneity, and steep dose fall-off, while maintaining all OAR doses within accepted stereotactic radiotherapy constraints. PTV: planning target volume; SBRT: stereotactic body radiotherapy; HI: homogeneity index; GI: gradient index

Structure	Parameter evaluated	Achieved dosimetric value	SBRT constraint status
PTV red (isolated para-aortic)	V100% (target volume)	99.19%	Optimal target coverage
	D2%	35.43 Gy	Acceptable
	HI	1.12	Acceptable homogeneity
	GI	1.20	Favorable dose gradient
Small bowel	Dmax (absolute max)	26.20 Gy	Within SBRT tolerance (<30 Gy)
Cauda equina	Dmax (absolute max)	6.95 Gy	Within SBRT tolerance (<25 Gy)
Right kidney	Mean dose	1.72 Gy	Within SBRT tolerance (<8.5 Gy)
Left kidney	Mean dose	0.10 Gy	Within SBRT tolerance (<8.5 Gy)

**Figure 6:**
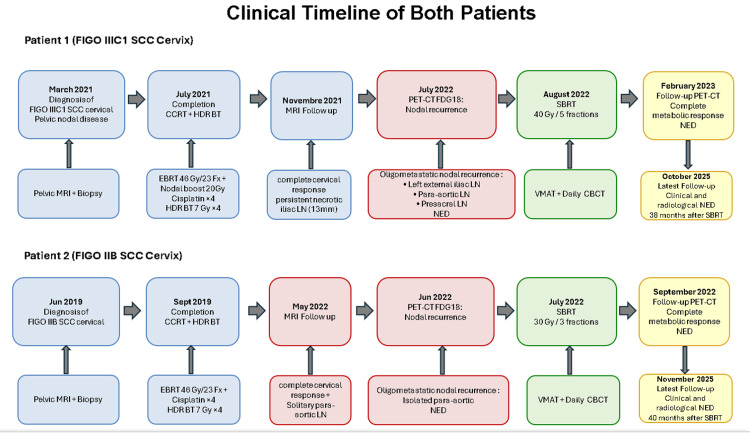
Clinical timeline and SBRT management of regional nodal recurrence in two patients with cervical cancer. Clinical timeline summarizing initial diagnosis, definitive chemoradiotherapy, regional nodal recurrence, salvage SBRT, and follow-up outcomes in both patients. CCRT: concurrent chemoradiotherapy; HDR-BT: high-dose-rate brachytherapy; NED: no evidence of disease; PET-CT: positron emission tomography-computed tomography; SBRT: stereotactic body radiotherapy; SCC: squamous cell carcinoma; VMAT: volumetric modulated arc therapy; FIGO: International Federation of Gynecology and Obstetrics; ^18^F-FDG: fluorine-18 fluorodeoxyglucose; CBCT: cone-beam computed tomography; LN: lymph node; EBRT: external beam radiotherapy

Clinical timelines summarizing the diagnostic course, primary treatment, disease-free interval, regional nodal recurrence, SBRT delivery, and follow-up outcomes for both patients are presented in Figure 6.

## Discussion

Regional nodal recurrence and rationale for SBRT

The management of isolated or limited regional nodal recurrence after definitive chemoradiotherapy for cervical cancer, particularly within previously irradiated pelvic or retroperitoneal fields, remains a major therapeutic challenge. Historically, recurrent cervical cancer was generally associated with a poor prognosis, and treatment strategies were largely limited to systemic therapy and symptom-directed care. However, the development of the oligometastatic and oligorecurrent disease concepts has transformed therapeutic decision-making, suggesting that selected patients with limited-volume recurrence may benefit from aggressive local treatment with curative intent [4-6].

In this setting, SBRT has emerged as an effective salvage modality, delivering highly conformal ablative doses with submillimetric precision while minimizing irradiation of surrounding OARs [2,6-8,15]. Several retrospective series and systematic reviews have reported favorable local control rates and acceptable toxicity profiles following SBRT for nodal recurrence in gynecological malignancies, including previously irradiated patients [2,8,9,13,15]. In the present report, both patients achieved complete metabolic responses after PET-CT-guided SBRT, supporting the feasibility and efficacy of this approach in carefully selected cases.

Histological confirmation was not obtained in either case. Although biopsy remains the gold standard for confirming recurrence, tissue sampling of deeply located pelvic or para-aortic lymph nodes may be technically challenging and associated with substantial procedural risks, particularly in previously irradiated patients because of fibrosis and proximity to major vascular structures [14]. In both patients, the diagnosis of recurrence was supported by highly suggestive ^18^F-FDG PET-CT findings, compatible clinical history, absence of alternative disease sites, and multidisciplinary tumor board consensus, supporting a high clinical probability of recurrence. The growing role of PET-CT in the detection, characterization, and treatment planning of nodal relapses in gynecological cancers has been highlighted in several studies, supporting its integration into salvage treatment strategies when histological confirmation is not feasible or would expose the patient to excessive risk [2,13,18]. This approach reflects contemporary clinical practice in selected patients with difficult-to-access nodal lesions.

Biological rationale and ablative efficacy

SBRT differs from conventional fractionated radiotherapy by delivering a high biological dose per fraction, resulting in direct tumor cell death as well as additional vascular and stromal effects [3,10,11]. High-dose irradiation induces endothelial apoptosis, microvascular dysfunction, and tumor necrosis, mechanisms that may overcome radioresistance associated with hypoxic tumor areas [10,11]. Furthermore, preclinical and translational studies suggest that SBRT may enhance anti-tumor immunity through immunogenic cell death, tumor antigen release, and activation of cytotoxic T-cell responses [11,12].

The effectiveness of SBRT is influenced by several factors, including delivered dose, target volume, tumor biology, and accuracy of image-guided treatment delivery [3,6]. Previous studies have reported high local control rates after SBRT for selected nodal recurrences in gynecological cancers, particularly when lesions are accurately delineated using metabolic imaging such as ^18^F-FDG PET-CT [2,13,18].

Comparative therapeutic strategies

For carefully selected patients with isolated or limited regional nodal recurrence, SBRT offers several advantages compared with alternative salvage approaches. Surgical lymphadenectomy may provide histological confirmation and potential local control but is often technically challenging in previously irradiated patients because of radiation-induced fibrosis, distorted anatomical planes, and an increased risk of vascular, ureteral, or lymphatic complications [14]. This limitation was illustrated in our second patient, in whom salvage para-aortic lymphadenectomy was attempted but ultimately abandoned because of dense retroperitoneal fibrosis and close adherence of the recurrent lymph node to the abdominal aorta.

Systemic therapy remains the standard treatment for disseminated recurrent cervical cancer according to contemporary guidelines [1,15]. However, its benefit in patients with isolated nodal relapse remains less well established, particularly when complete local ablation of all detectable disease can be achieved. Moreover, systemic treatments may be associated with cumulative hematologic, renal, and neurological toxicities that can negatively affect quality of life [7,15].

Conventional re-irradiation using standard fractionation is another potential option but is frequently limited by prior radiation exposure and the risk of exceeding tolerance doses of adjacent OARs, potentially leading to severe late toxicity [14]. In contrast, SBRT enables the delivery of highly conformal ablative doses with steep dose gradients and precise image guidance, thereby maximizing target coverage while minimizing irradiation of surrounding normal tissues [2,6,14,15].

Notably, neither patient received chemotherapy, immunotherapy, targeted therapy, or other systemic treatment before or after SBRT. The complete metabolic responses and durable disease control observed in both cases, therefore, reflect the efficacy of local ablative treatment alone. These observations support the concept that selected patients with isolated regional nodal recurrence may benefit from metastasis-directed therapy, potentially delaying or avoiding systemic treatment and its associated toxicities [1,7,15].

Clinical evidence supporting SBRT

The role of SBRT in limited-volume regional nodal recurrence or limited metastatic disease has been supported by prospective and retrospective evidence. The randomized phase II SABR-COMET trial demonstrated improved overall survival with the addition of stereotactic ablative radiotherapy in selected patients with oligometastatic cancer across multiple primary tumor types, although cervical cancer represented only a minority of enrolled patients [16,17]. Therefore, these results should be interpreted as supporting the broader concept of metastasis-directed therapy rather than providing cervical cancer-specific evidence.

In cervical cancer, several retrospective series have reported favorable outcomes with SBRT for limited-volume regional nodal recurrence. Choi et al. evaluated SBRT for para-aortic nodal recurrence after definitive treatment and reported durable local control with acceptable toxicity rates [2]. Similarly, Jereczek-Fossa et al. and Fodor et al. demonstrated that PET-CT-guided SBRT achieved high local control rates in selected patients with nodal recurrences, including previously irradiated regions [8,18].

A summary of representative studies evaluating SBRT for regional nodal recurrence in gynecological cancers is presented in Table 3.

**Table 3:** Representative studies evaluating SBRT for oligometastatic or regional nodal recurrence in gynecological malignancies. Selected studies reporting outcomes of SBRT for isolated or limited nodal recurrence in gynecological cancers, demonstrating favorable local control and acceptable toxicity profiles. SBRT: stereotactic body radiotherapy; SABR: stereotactic ablative radiotherapy; Gy: Gray; OS: overall survival; PFS: progression-free survival; ^18^F-FDG PET-CT: fluorine-18 fluorodeoxyglucose positron emission tomography-computed tomography

Study	Number of patients	Site	Dose/fractions	Local control	Key findings
Choi et al. [2]	30	Para-aortic lymph nodes	33-45 Gy/3 fractions	70%-80%	SBRT represents an effective salvage option for isolated para-aortic nodal recurrence.
Fodor et al. [18]	58 (35 treated with SBRT)	Nodal oligometastases	30-40 Gy/3-5 fractions	85%-90%	^18^F-FDG PET-CT-guided SBRT achieved high local control with acceptable toxicity.
Jereczek-Fossa et al. [8]	69	Lymph node metastases	30-45 Gy/3-6 fractions	~85%	SBRT was feasible and safe, including in previously irradiated pelvic fields.
Palma et al. (SABR-COMET) [16,17]	99	Mixed oligometastatic sites	Variable/individualized	Improved OS and PFS	SBRT significantly improved OS and PFS compared with standard care alone.
Milano et al. [19]	121	Oligometastatic sites	Variable/hypofractionated	Durable long-term control	Confirmed long-term safety and sustained disease control following SBRT.

PET-CT role and response assessment

In both patients presented here, SBRT resulted in complete metabolic responses, supporting the role of functional imaging throughout the treatment pathway. ^18^F-FDG PET-CT contributes not only to accurate identification of active nodal disease and exclusion of disseminated metastases but also to treatment planning and evaluation of metabolic response after therapy [2,13,18]. The integration of metabolic imaging into SBRT workflows may improve target delineation accuracy and facilitate early assessment of treatment efficacy [18].

Safety profile and future perspectives

Despite the delivery of ablative doses, SBRT is generally well tolerated when performed using modern image-guided radiotherapy techniques and strict OAR constraints [2,8,15]. Reported rates of grade ≥3 toxicity remain low in published series of gynecological nodal recurrences, generally ranging between 5% and 10% [8,15].

In our experience, no severe acute toxicity was observed. One patient developed mild late gastrointestinal toxicity (grade 1 intermittent diarrhea), likely related to cumulative radiation exposure from previous pelvic chemoradiotherapy and subsequent para-aortic SBRT. Symptoms were managed conservatively without hospitalization or treatment interruption.

These findings emphasize the importance of meticulous treatment planning, PET-CT-based target delineation, daily image guidance, and rigorous dosimetric evaluation, particularly in previously irradiated patients [2,14,18]. VMAT-based SBRT enables highly conformal dose delivery with rapid dose fall-off, allowing adequate target coverage while limiting exposure to bowel, kidneys, spinal canal, and other critical structures [3,14,15].

However, current evidence remains limited by the predominance of retrospective studies, heterogeneous patient selection criteria, and variability in dose-fractionation schedules [8,9,15]. Optimal indications, re-irradiation constraints, and the definition of candidates most likely to benefit from ablative treatment remain under investigation [5,6,15].

Beyond local control, growing evidence suggests that SBRT may enhance systemic immune responses through modulation of the tumor microenvironment and immunogenic cell death pathways [10-12]. Combination strategies integrating SBRT with systemic therapies, including immune checkpoint inhibitors, represent an emerging area of investigation in recurrent cervical cancer [15-17].

Limitations

This report is limited by its retrospective design, the small sample size of two patients, and the absence of histological confirmation of recurrence, which preclude definitive conclusions regarding efficacy and toxicity. Nevertheless, follow-up durations reached 38 and 40 months after SBRT for Patients 1 and 2, respectively, allowing assessment of intermediate-term local control, durability of metabolic response, and treatment-related toxicity outcomes. Despite these encouraging results, longer follow-up and larger prospective studies are required to better define long-term disease control, survival outcomes, optimal patient selection, and late toxicity profiles. These cases nevertheless illustrate the feasibility of PET-CT-guided SBRT as a salvage treatment option for carefully selected patients with isolated regional nodal recurrence after prior chemoradiotherapy and support further prospective evaluation [5,8,15-17,19].

## Conclusions

SBRT appears to be a feasible, safe, and effective salvage treatment for selected patients presenting with limited-volume regional nodal recurrence of cervical cancer after prior definitive chemoradiotherapy. PET-CT-guided VMAT-based SBRT achieved complete metabolic response and durable local control in both patients while maintaining acceptable toxicity profiles. Although larger prospective studies are required, these cases support the growing role of metastasis-directed therapy and curative-intent local treatment strategies for carefully selected patients with limited-volume recurrent cervical cancer.
